# Supplementary material for: Perturbation of amygdala-cortical projections reduces ensemble coherence of palatability coding in gustatory cortex
Source: eLife. 2021 May 21;10:e65766. doi: 10.7554/eLife.65766 (PMC8139825; doi:10.7554/eLife.65766)
Supplement: Transparent reporting form [file elife-65766-transrepform.pdf]

Westbrook Centre
Milton Road
Cambridge CB4 1YG, UK

P 01223 855340
W elifesciences.org
T @elife

eLife's transparent reporting form

We encourage authors to provide detailed information *within their submission* to facilitate the interpretation and replication of experiments. Authors can upload supporting documentation to indicate the use of appropriate reporting guidelines for health-related research (see EQUATOR Network), life science research (see the BioSharing Information Resource), or the ARRIVE guidelines for reporting work involving animal research. Where applicable, authors should refer to any relevant reporting standards documents in this form.

If you have any questions, please consult our Journal Policies and/or contact us: editorial@elifesciences.org.

Sample-size estimation

- You should state whether an appropriate sample size was computed when the study was being designed
- You should state the statistical method of sample size computation and any required assumptions
- If no explicit power analysis was used, you should describe how you decided what sample (replicate) size (number) to use

Please outline where this information can be found within the submission (e.g., sections or figure legends), or explain why this information doesn't apply to your submission:

> Based on 20 years of prior experiments involving recordings of cortical taste response, we have the expertise to provide assurance that a population of 140 recorded cortical neurons provides a statistical power with which we can reveal meaningful results. Accordingly, no explicit power analysis was necessary.

Replicates

- You should report how often each experiment was performed
- You should include a definition of biological versus technical replication
- The data obtained should be provided and sufficient information should be provided to indicate the number of independent biological and/or technical replicates
- If you encountered any outliers, you should describe how these were handled
- Criteria for exclusion/inclusion of data should be clearly stated
- High-throughput sequence data should be uploaded before submission, with a private link for reviewers provided (these are available from both GEO and Array Express)

Please outline where this information can be found within the submission (e.g., sections or figure legends), or explain why this information doesn't apply to your submission:

> The sample size for each analysis is included in the Results section and figures. As noted in the text (Results and Discussion), the basic finding of this research both replicates the taste dynamics demonstrated in many prior publications and reveals novel understanding regarding how the dynamic is generated.

eLife Sciences Publications, Ltd is a limited liability non-profit non-stock corporation incorporated in the State of Delaware, USA, with company number 5030732, and is registered in the UK with company number FC030576 and branch number BR015634 at the address Westbrook Centre, Milton Road, Cambridge CB4 1YG.

Statistical reporting

- Statistical analysis methods should be described and justified
- Raw data should be presented in figures whenever informative to do so (typically when N per group is less than 10)
- For each experiment, you should identify the statistical tests used, exact values of N, definitions of center, methods of multiple test correction, and dispersion and precision measures (e.g., mean, median, SD, SEM, confidence intervals; and, for the major substantive results, a measure of effect size (e.g., Pearson's r, Cohen's d)
- Report exact p-values wherever possible alongside the summary statistics and 95% confidence intervals. These should be reported for all key questions and not only when the p-value is less than 0.05.

Please outline where this information can be found within the submission (e.g., sections or figure legends), or explain why this information doesn't apply to your submission:

> The analyses included are clearly announced and described in the Methods and Results sections, including the types of analyses and stats (e.g., t-, f-values etc.). The criterion for significance was set at 0.05. For analysis involves multiple comparisons, a more stringent criterion of significance was applied (see the section of the palatability correlation analysis). It is worth noting that our tested hypotheses were both highly specific and directional (i.e., not simply "perturbations will change things") – a fact that indirectly increases the conservatism of the tests.

(For large datasets, or papers with a very large number of statistical tests, you may upload a single table file with tests, Ns, etc., with reference to sections in the manuscript.)

Group allocation

- Indicate how samples were allocated into experimental groups (in the case of clinical studies, please specify allocation to treatment method); if randomization was used, please also state if restricted randomization was applied
- Indicate if masking was used during group allocation, data collection and/or data analysis

Please outline where this information can be found within the submission (e.g., sections or figure legends), or explain why this information doesn't apply to your submission:

> To increase the statistical power and meaningful comparisons between groups, we have used within-subject designs. These facts are clearly stated in the Results and Methods sections.

Additional data files ("source data")

- We encourage you to upload relevant additional data files, such as numerical data that are represented as a graph in a figure, or as a summary table
- Where provided, these should be in the most useful format, and they can be uploaded as "Source data" files linked to a main figure or table
- Include model definition files including the full list of parameters used
- Include code used for data analysis (e.g., R, MatLab)
- Avoid stating that data files are "available upon request"

Please indicate the figures or tables for which source data files have been provided:

eLife Sciences Publications, Ltd is a limited liability non-profit non-stock corporation incorporated in the State of Delaware, USA, with company number 5030732, and is registered in the UK with company number FC030576 and branch number BR015634 at the address Westbrook Centre, Milton Road, Cambridge CB4 1YG.

Our electrophysiology datasets are saved in a hierarchical data format (HDF5) and the files are hosted on a university-wide network share managed by Library and Technology Services (LTS) at Brandeis University. Each HDF5 file contains electrophysiology recordings, sorted spikes, single-neuron and population-level analyses. These files are thus very large and difficult to be hosted on a general-purpose file share platform. That said, the corresponding author, Donald Katz (dbkatz@brandeis.edu), will happily supply the data upon request by granting the permission to access the Katz-lab share at files.brandeis.edu.

eLife Sciences Publications, Ltd is a limited liability non-profit non-stock corporation incorporated in the State of Delaware, USA, with company number 5030732, and is registered in the UK with company number FC030576 and branch number BR015634 at the address Westbrook Centre, Milton Road, Cambridge CB4 1YG.
